# Macrophage P2Y_6_ receptor signalling as a key mediator and therapeutic target in atherosclerosis

**DOI:** 10.1007/s11302-025-10083-w

**Published:** 2025-03-12

**Authors:** Aida Collado, Zhichao Zhou

**Affiliations:** Division of Cardiology, Department of Medicine Solna, Karolinska Institutet, Solna, Stockholm 17177 Sweden

**Keywords:** P2Y_6_ receptor, Macrophage, Atherosclerosis

## Abstract

Atherosclerosis, a chronic inflammatory disease driven by lipid deposition and immune cell activation, remains a leading cause of cardiovascular morbidity and mortality. Emerging evidence highlights the role of purinergic signalling in atherogenesis, particularly the P2Y_6_ receptor in macrophages [1]. Using RNA sequencing, proteomics, expression and functional validation in cells, mouse models and human materials, this study provides comprehensive mechanistic insights into how macrophage P2Y_6_ receptors contribute to foam cell formation and plaque development through the phospholipase Cβ (PLCβ)/store-operated Ca^2+^ entry/calreticulin/scavenger receptor A (SR-A) pathway. Furthermore, the study identifies thiamine pyrophosphate (TPP) as a potent P2Y_6_ receptor antagonist, effectively inhibiting foam cell formation and reducing plaque burden in atherosclerotic mice, without inducing toxicity. These findings establish P2Y_6_ receptors as promising therapeutic targets in atherosclerosis and introduce TPP as a potential clinical candidate for intervention.

## Commentary

Atherosclerosis, characterized by lipid deposition and chronic inflammation, is a leading cause of coronary heart disease, cerebral infarction and peripheral vascular disease. When lesions obstruct the arterial lumen, the affected tissues or organs may experience ischemia or necrosis. Macrophages, a central component of atheromatous plagues, play a crucial role in the initiation and progression of atherosclerosis by taking up the oxidized low-density lipoprotein (ox-LDL) via scavenger receptors, leading to foam cell formation [2]. Purinergic dysfunction contributes to cardiovascular disease, including atherosclerosis. Studies have shown that several purinergic receptors, such as A_3_, P2X_4_, P2X_7_, P2Y_1_, P2Y_6_, and P2Y_12_ receptors, contribute to the initiation and progression of atherosclerosis and targeting them has shown therapeutic potential [3, 4]. However, the precise role of P2Y_6_ receptors in macrophages, as well as the mechanisms by which P2Y_6_ receptors influence foam cell formation and atherogenesis remain unclear.

A recent study published in the *European Heart Journal* [1] identified macrophages as the predominant site of P2Y_6_ receptor activation. The authors analyzed data from a public database and found that P2Y_6_ receptor mRNA levels were elevated in human atherosclerotic plaques, with the most abundant expression in macrophages from human atherosclerotic arteries. The upregulation of P2Y_6_ protein levels in macrophages was further observed in both the aortic roots of high-fat diet (HFD)-fed LDLR^−/−^ mice and human carotid atherosclerotic plaques. To further confirm the macrophage-specific role of the P2Y_6_ receptor, the authors generated macrophage-specific P2Y_6_ receptor knockout, HFD-fed LDLR^−/−^ mice. These mice developed smaller plaques and exhibited reduced lesion formation in the aortic root, reinforcing a functional link between macrophage P2Y_6_ receptor expression and atherosclerosis progression. This strong functional evidence observed in a 12-week HFD-fed LDLR^−/−^ mice differs from previous studies that used prolonged high cholesterol treatment or severe inflammatory atherosclerotic model [5, 6]. This suggests that P2Y_6_ receptors may play distinct roles at different stages of atherosclerosis development. The macrophage P2Y_6_ receptor-mediated actions may be more dominant in the early stages of lesion development, while P2Y_6_ receptors in other cell types may become more relevant in later disease stages.

Using loss-of-function genetic approaches and multi-omics analyses, the authors revealed comprehensive mechanistic insights into P2Y_6_ receptor-mediated downstream signalling pathways. They found that activation of the P2Y_6_ receptor occurs through its canonical G-protein partner Gαq/11 and PLCβ, triggering Ca^2+^ release from the endoplasmic reticulum. This, in turn, stimulates STIM dissociation from calreticulin and its translocation to the plasma membrane, leading to ORAI1 channel activation and Ca^2+^ influx. Proteomic analysis followed by functional experiments further revealed that P2Y_6_ receptor-mediated Ca^2+^ disruption enhances the interaction between calreticulin and SR-A, a key macrophage scavenger receptor involved in foam cell formation. This interaction facilitates ox-LDL uptake, promotes cholesterol accumulation in macrophages, and accelerates atherosclerosis progression. These findings expand our understanding of foam cell formation mechanisms, highlighting how an imbalance between the STIM-calreticulin complex and the calreticulin-SR-A complex, regulated by P2Y_6_ receptor signalling, drives disease progression. Additionally, UDP-mediated nitric oxide signalling was observed to be impaired in P2Y_6_ receptor knockout mice [7]. Given that nitric oxide plays a key role in both purinergic and Ca^2+^ signalling, further investigations into its involvement in the P2Y_6_ receptor-mediated Ca^2+^ release in macrophages could provide deeper insights into complex networks contributing to atherosclerosis.

An equally compelling finding of this study is the identification of TPP as an effective P2Y_6_ receptor antagonist for the treatment of atherosclerosis. Using a high-throughput Glide docking pipeline for drug repurposing, the authors screened existing drug libraries and discovered TPP’s potential. TPP reduced the foam cell formation in vitro cell model, while the delivery of TPP to HFD-fed LDLR^−/−^ mice by gavage produced dose-dependent effects. TPP significantly decreased plaque formation, lipid deposition and pro-inflammatory factors, including IL-1β, TNF-α, IL-6 and IL-8, via the inhibition of P2Y_6_ receptors, without causing obvious toxic effects. However, the study did not include pharmacokinetic analyses, leaving questions about optimal dosing, the most effective route of administration, potential off-target effects and long-term safety. Further investigations are needed to address these concerns. Taken together, these findings highlight TPP as a promising P2Y_6_ receptor antagonist, positioning it as a potential clinical candidate for atherosclerosis treatment, given its prior approval for other indications.

Overall, this study provides comprehensive mechanistic insights into how macrophage P2Y_6_ receptor-mediated PLCβ/store-operated Ca^2+^ entry/calreticulin/SR-A signalling pathways contribute to atherosclerosis. It also highlights the therapeutic potential of targeting macrophage P2Y_6_ receptors using TPP in an atherosclerotic mouse model. While the downstream signalling of P2Y_6_ receptors is clearly delineated, the primary source of UTP and UDP responsible for macrophage P2Y_6_ receptor activation in this model remains to be identified. Targeting purinergic receptors has shown promising outcomes in atherosclerotic mouse models. Given the diversity of purinergic receptor subtypes, such as P2X_7_ and P2Y_6_ in macrophages [1, 8], as well as A_3_, P2X_4_, P2X_7_, P2Y_1_, P2Y_6_ and P2Y_12_ in other cell types [3, 4, 9], the question of which purinergic receptor(s) to target in which cells for a synergistic therapeutic effect remains unexplored. Additionally, whether P2Y_6_ receptor inhibition or blockade of other purinergic receptors could be effectively combined with existing lipid-lowering therapies—such as statins, PCSK9 inhibitors, and IL-1β blockers—to enhance therapeutic efficacy warrants further investigation. By elucidating the role of purinergic signalling in macrophage-driven atherosclerosis and highlighting the therapeutic potential of targeting P2Y_6_ receptors, this study provides valuable insights that could guide future clinical interventions for atherosclerosis treatment.

## Data Availability

No datasets were generated or analysed during the current study.
